# Introduction to the Special Issue on Improving Early Childhood Nurturing Care to Support Childhood Development and Adult Health

**DOI:** 10.3390/children10050806

**Published:** 2023-04-29

**Authors:** Hermano Alexandre Lima Rocha

**Affiliations:** Department of Community Health, Federal University of Ceará, Fortaleza 60430-235, CE, Brazil; hermano@ufc.br; Tel.: +55-(85)-3366-8044

**Background:** Child development is an ongoing process that occurs from birth to adolescence and is shaped by various factors, such as genetics, the environment, and experiences. As present, there are many emerging trends in child development, including improvements in health and education, but there are also new challenges related to nutrition, responsive caregiving, security and safety, and health; many of these have been aggravated by the COVID-19 pandemic [1].

To address these challenges, various initiatives have been launched by governments, non-governmental organizations, and international organizations. For instance, the World Health Organization (WHO) developed the Nurturing Care Framework for Early Childhood Development, a global strategy to promote optimal child development and well-being. It emphasizes the importance of the holistic, nurturing care of young children to ensure their healthy physical, cognitive, social, and emotional development [2].

The framework is based on the latest scientific evidence, which shows that early childhood experiences are critical in shaping lifelong health, learning, and behavior. It recognizes that children need to be cared for by their parents, caregivers, and communities to achieve their full potential. The framework outlines five key components of nurturing care: health, nutrition, responsive caregiving, safety and security, and early learning. It is a critical tool for promoting optimal child development and worldwide well-being. Emphasizing the importance of nurturing care in the critical early years of life can help break cycles of poverty, inequality, and poor health outcomes. It can also guide policies and programs that support early childhood development across all sectors and promote multi-sectoral collaboration and community engagement [3].

This Special Issue features papers exploring topics that expand our understanding of nurturing care for children across all five key components. So far, 19 articles have been published, and the twentieth one has been submitted, demonstrating the volume of research dedicated to this topic. Among these articles, the work by Bang et al. studied the factors associated with diabetes among young people [4]. Stegariu et al. revealed a linear relationship between intelligence and body schema, and we can use the first one to predict the evolution of the second one [5]. In addition, Martin-Rodriguez shed light on the problem of childhood obesity and the potential for them to become adults with chronic diseases in the future [6], while Carlos-Vivas et al. studied the higher SBP, DBP, triceps skinfold, and abdominal circumference values among overweight/obese adolescents of both genders compared to those with a normal weight [7]. Finally, Kuse et al. noted that anemia is still a problem among Ethiopian children [8]. In the domain of responsive caregiving, my research group demonstrated that positive parenting behaviors are robustly associated with better outcomes across developmental domains among Brazilian children [9]. Pinero-Pinto et al. introduced the Spanish version of “This is my baby”, which is an interview that measures the acceptance, commitment, and awareness of the influence of parents on their babies [10].

The research groups of Puerto-Golzarri and Carpinelli brilliantly represented the domain of safety and security. The first work explored the importance of parenting styles with authoritarian characteristics in the occurrence of aggressive behavior in children [11], while the second one presented the result of an important intervention that aimed to reinforce the skills of the operators that prevent and combat adverse childhood experiences (ACEs), demonstrating that this intervention helped in the identification of cases of chronic and severe exposure to ACEs [12].

Health and early learning domains were also well represented in this collection. Islam et al. and Gmmash et al. studied factors associated with health services provided to children in Saudi Arabia and India [13,14]. Rojo-Ramos et al., Soares et al., Chan et al., Lu et al., and Agrawal et al. presented aspects that represent the importance of the educational environment during childhood for adequate nurturing care [15,16,17,18,19]. For example, Lee’s group studied the effects of music intervention on fetal education among pregnant women, demonstrating that the fetus receives external sounds through hearing, and a pregnant woman singing her fetus can stabilize the frequency of fetal movement, promote the health of herself and the fetus, and establish maternal–fetal bonding [20]. In terms of the combined health and education domains, Enríquez-del-Castillo et al. demonstrated that a six-week training program of moderate intensity improved children’s physical fitness [21]. Pereira et al. analyzed nurses’ evaluation of the health education practice for children and parents in Portugal, which is not often discussed in Portuguese-speaking countries [22].

This collection aimed to bring new evidence for the WHO’s Nurturing Care Framework due to the current research gaps in designing efficient interventions for improving children’s development [23]. Although there are already reviews with proposals for multiple interventions for nurturing care [24] and comprehensive evaluations of domains in specific contexts [25,26,27,28], more research is still needed to build consistent public policies for improving children’s development. We hope this collection will be another pillar for achieving this.
